# Impact of coinfection status and comorbidity on disease severity in adult emergency department patients with influenza B

**DOI:** 10.1111/irv.12907

**Published:** 2021-09-17

**Authors:** Alexander J. Zapf, Justin Hardick, Breana McBryde, Lauren M. Sauer, Katherine Z. J. Fenstermacher, Erin P. Ricketts, Yi‐Chin Lin, Kuan‐Fu Chen, Yu‐Hsiang Hsieh, Andrea Dugas, Kathryn Shaw‐Saliba, Andrew Pekosz, Charlotte A. Gaydos, Richard E. Rothman, Emergency Department National Network Influenza Investigators

**Affiliations:** ^1^ Department of International Health Johns Hopkins Bloomberg School of Public Health Baltimore Maryland USA; ^2^ Division of Infectious Diseases Johns Hopkins University School of Medicine Baltimore Maryland USA; ^3^ Department of Emergency Medicine Johns Hopkins University School of Medicine Baltimore Maryland USA; ^4^ Department of Emergency Medicine Chang Gung Memorial Hospital Keelung Taiwan; ^5^ Clinical Informatics and Medical Statistics Research Center Chang Gung University Taoyuan Taiwan; ^6^ W. Harry Feinstone Department of Molecular Microbiology and Immunology Johns Hopkins Bloomberg School of Public Health Baltimore Maryland USA

**Keywords:** coinfection, comorbidity, disease severity, emergency service, hospital, influenza B, influenza, human

## Abstract

**Background:**

Influenza B accounts for approximately one fourth of the seasonal influenza burden. However, research on the importance of influenza B has received less attention compared to influenza A. We sought to describe the association of both coinfections and comorbidities with disease severity among adults presenting to emergency departments (ED) with influenza B.

**Methods:**

Nasopharyngeal samples from patients found to be influenza B positive in four US and three Taiwanese ED over four consecutive influenza seasons (2014–2018) were tested for coinfections with the ePlex RP RUO panel. Multivariable logistic regressions were fitted to model adjusted odds ratios (aOR) for two severity outcomes separately: hospitalization and pneumonia diagnosis. Adjusting for demographic factors, underlying health conditions, and the National Early Warning Score (NEWS), we estimated the association of upper respiratory coinfections and comorbidity with disease severity (including hospitalization or pneumonia).

**Results:**

Amongst all influenza B positive individuals (*n* = 446), presence of another upper respiratory pathogen was associated with an increased likelihood of hospitalization (aOR = 2.99 [95% confidence interval (95% CI): 1.14–7.85, *p* = 0.026]) and pneumonia (aOR = 2.27 [95% CI: 1.25–4.09, *p* = 0.007]). Chronic lung diseases (CLD) were the strongest predictor for hospitalization (aOR = 3.43 [95% CI: 2.98–3.95, *p* < 0.001]), but not for pneumonia (aOR = 1.73 [95% CI: 0.80–3.78, *p* = 0.166]).

**Conclusion:**

Amongst ED patients infected with influenza B, the presence of other upper respiratory pathogens was independently associated with both hospitalization and pneumonia; presence of CLD was also associated with hospitalization. These findings may be informative for ED clinician's in managing patients infected with influenza B.

## 1 INTRODUCTION

Annually, seasonal influenza epidemics result in an estimated 290 000 to 650 000 deaths globally^1^ and account for between 9 and 45 million cases of illnesses annually in the United States alone.^2^ Seasonal epidemics are caused by two types of influenza viruses: influenza A and B.^3^ Historically, research has largely focused on influenza A due to its genetic variability, seasonal dominance, and pandemic potential.^4^, ^5^, ^6^, ^7^

Influenza B viruses primarily infect humans without continuous circulation in animal reservoirs and undergo slower genetic variability over time.^8^ Thus, their pandemic potential is perceived to be less than influenza A.^7^ However, influenza B viruses have the potential to predominate seasonal circulation as demonstrated during the 2019–2020 season^2^ and have been shown to be associated with severe disease, in both pediatric and young adult populations.^5^, ^9^, ^10^

The presence of comorbidities, including cardiovascular diseases and chronic lung diseases, represent known risk factors for severe influenza complications,^2^ but these risk factors have primarily been derived from studies of patients with influenza A.^7^, ^11^, ^12^, ^13^ In addition, the presence of coinfections with bacterial and/or viral pathogens has been found to be associated with increased disease severity, from studies of individuals with influenza A.^14^, ^15^, ^16^ Systematic epidemiologic analyses regarding the association of comorbidity and coinfections in those infected influenza B have been relatively limited.^5^, ^7^, ^17^

In this investigation, we retrospectively evaluated a large biorepository of nasopharyngeal specimens (NPs) taken from ED patients found to be influenza B positive. Specimens and corresponding clinical data were collected from four US emergency departments (ED) and three Taiwanese ED over four consecutive seasons, 2014–2018. The NPs were tested for the presence of coinfections, and the clinical and laboratory data were evaluated to determine potential associations of other upper respiratory pathogens and comorbidities with disease severity in those with influenza B.

## 2 METHODS

### 2.1 Ethical clearance and approval by institutional review boards

The studies were reviewed and approved by all participating institutions' Institutional Review Boards (IRB). Johns Hopkins University School of Medicine IRB approved protocols: IRB00135664, IRB00041233, IRB00141101, IRB00052743, and IRB00091667.

### 2.2 Setting, study participants, and data collection

NPs and clinical data for this analysis were collected from patients who presented to the EDs of four US hospitals and three hospitals in Taiwan over four consecutive influenza seasons from 2014–2018. All subjects were part of two federally funded parent studies in which ED patients with suspected influenza were prospectively enrolled. US sites included The Johns Hopkins Hospital (JHH) in Baltimore, MD; Maricopa Medical Center in Phoenix, AZ; Olive View‐UCLA Medical Center in Sylmar, CA; Truman Medical Center in Kansas City, MO); the Taiwan sites were the Chang Gung Memorial Hospitals (CGMH) in Taipei, Keelung, and Linkou, Taiwan.

Patients presenting to the study EDs with symptoms indicative of a respiratory infection were tested for influenza and respiratory syncytial virus (RSV) using the Cepheid GeneXpert Flu/RSV assay (Cepheid, Sunnyvale, CA) in the associated hospital clinical laboratory. Study design and methods for testing patients in the four US sites were previously published^18^; all subjects from Taiwan were enrolled and tested by dedicated study coordinators who approached patients with confirmed and/or influenza‐like illnesses with a protocol modified slightly from the one used in US sites. For all subjects who tested positive for influenza, a structured data collection form was completed by trained research coordinators using information from the electronic health records (EHR). Data gathered included demographic characteristics, clinical information (including comorbidities), and clinical outcomes (including oxygen supplementation, disposition, length of hospital stay, and pneumonia diagnosis, as determined by the attending physician through radiological interpretation). Each patient was assigned a de‐identified number. All de‐identified data were entered into a secure Research Electronic Data Capture database, and the dataset underwent rigorous quality control measures to ensure accuracy.

### 2.3 Identification and classification of coinfections

**FIGURE 1:**
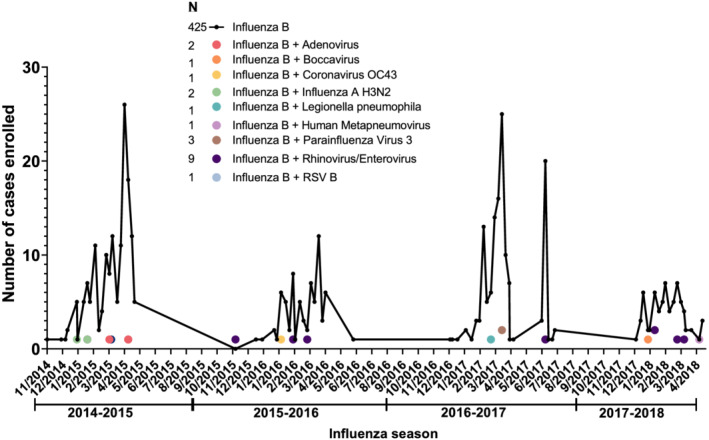
Number of enrolled cases per month for influenza B infections (*n* = 425) and influenza B coinfections with other respiratory pathogens (*n* = 21) across influenza seasons from 2014–15 to 2017–18 (*n* = 446); combined data from all seven sites. Connected black dots represent influenza B infections. Colored dots represent coinfections by respiratory pathogen type. RSV, respiratory syncytial virus

The influenza B positive NPs from the enrollment visit were retrospectively analyzed for coinfections with other respiratory pathogens using the Genmark ePlex RP RUO cartridge (Genmark Diagnostics, Carlsbad, CA) according to the manufacturer's instructions. Pathogens that are detectable by this assay include adenovirus; coronaviruses HKU1, NL63, OC43, 229E, MERS; human metapneumovirus; influenza A, A/H1N1, A/H1N1pdm 2009, A/H3N2; influenza B; parainfluenza 1–4; rhinovirus/enterovirus; RSV A/B; *Bordetella pertussis*, *Chlamydia pneumoniae*, *Legionella pneumophila*, *Mycoplasma pneumoniae*. Coinfections were aggregated into a binary indicator to reduce statistical imprecision associated with a small number of observations for individual pathogens (Figure 1).

### 2.4 Data analysis

Assessment of coinfections with 17 common viral and four atypical bacterial respiratory pathogens represented the primary explanatory variable for severe influenza B disease in our analyses. All models were adjusted for demographic factors and underlying health conditions according to the Centers for Disease Control and Prevention (CDC) classification of individuals at increased risk of influenza complications.^2^, ^19^ To control for clinical risk upon presentation to the ED, we adjusted for the National Early Warning Score for acutely ill patients (NEWS)^20^ as a validated risk predictor for respiratory infections.^21^ Classifications based on the NEWS range between 0 and greater 7.^20^, ^21^ Hospitalization and pneumonia diagnosis were used here as our outcome measure for disease severity; each has previously been reported as measure of disease severity,^12^, ^22^, ^23^ and both were systematically collected across all years and all study sites. Disposition and pneumonia diagnosis were dichotomized (admitted v. discharged patients and pneumonia v. no pneumonia diagnosis as determined by radiological findings).

### 2.5 Univariate statistics and bivariate analyses

Characteristics of the study population were assessed using summary statistics, while distributions were inspected via Q‐Q‐plots and Shapiro–Wilk test for normality.^24^ Categorical variables and outcomes by coinfection status were evaluated using Pearson's Chi‐square and Fisher exact statistics. Differences in continuous variables and ordinal scores were evaluated via Wilcoxon rank‐sum tests and unpaired *t* tests for unequal variance. Unadjusted, bivariate associations between the two severity outcomes and individual predictors were explored by computing correlation coefficients, scatterplots, and simple logistic regressions.

### 2.6 Multivariable logistic regression models

All baseline models were adjusted for age, gender, race, and ethnicity. The covariate for age was centered at the average of 43.2 years for better interpretability of adjusted estimates. The indicators for race and ethnicity were coded with White and non‐Hispanic/non‐Latino participants constituting the reference group, respectively. Separate multivariable logistic regressions for each of the two severity outcomes were built by iteratively including predictor variables and assessing model fit improvement via likelihood ratio test (LRT) statistics. These manually fitted models were validated by using computational algorithms for forward and backward selection based on Aikaike Information Criterion (AIC) values as well as best subset variable selection algorithms.^25^ Models for hospitalization were fitted using linear indicators for NEWS and underlying health conditions. For the pneumonia models, binary indicators with cut‐offs at NEWS = 5 and one underlying health condition were selected based on superior model performance and previous literature.^26^ To adjust for heterogeneity across study sites, robust variance estimates^27^ based on Huber^28^ and White^29^ were used.

Interactions between coinfection status and other severity predictors were inspected by testing interaction terms and assessing changes in model fit via LRT statistics. Adjusted probabilities for the two severity outcomes by coinfection status were predicted from the fitted main models, holding other predictors constant at their mean.

### 2.7 Comorbidity analysis

A secondary analysis on the impact of individual comorbidities on disease severity was conducted. Respiratory conditions, including chronic obstructive pulmonary disease, asthma, cystic fibrosis, tuberculosis, and emphysema, were combined into one predictor as chronic lung diseases (CLD). Adjusted probabilities were estimated analogously to the primary models from the fitted regression models.

### 2.8 Model validation

Goodness of model fit was assessed using Hosmer‐Lemeshow test statistics,^30^ and collinearity of predictors was inspected through variance inflation factors (VIF). As test statistics for both models were non‐significant (*p* > 0.05) and VIF consistently ranked below VIF = 5, adequate fit for the estimated models can be assumed. Validation of model predictions was performed through *k‐fold* cross‐validation techniques by iteratively leaving out one observation.

All statistical analyses were performed in Stata 15.1 (StataCorp LLC), and figures were refined using Prism9 (GraphPad software).

## 3 RESULTS

### 3.1 Summary of the study population

A summary of the study population characteristics, comorbidities, and major disease severity outcomes is displayed in Table 1. A total of 446 ED patients with influenza B virus infection were identified across all sites and seasons. The majority (53.1% [237/446]) of influenza B cases were from JHH followed by CGMH, which had 17.5% (78/446) of cases. Across the four seasons, most influenza B cases were identified during the 2014–15 (34.8% [155/446]) and 2016–17 (31.8% [142/446]) seasons. The relative frequency of coinfections across all seasons was 47 per 1000 (21/446) influenza B patients. The most frequent coinfections observed were rhinovirus/enterovirus (42.9% [9/21]), parainfluenza virus (14.3% [3/21]), and adenovirus (9.5% [2/21]), but there was no obvious temporal pattern of coinfections observed across seasons (Figure 1).

**TABLE 1:** Summary of the study population characteristics, comorbidities, and clinical severity outcomes (*n* = 446)

	Total (*n* = 446)	Influenza B (*n* = 425)	Influenza B + coinfection (*n* = 21)	*P* value*
**Study site**				0.07
Johns Hopkins Hospital—no. (%)	237 (53.0)	230 (54.0)	7 (33.0)	
Chang Gung Memorial Hospitals (Taiwan)—no. (%)	78 (17.0)	71 (17.0)	7 (33.0)	
Maricopa Medical Center—no. (%)	48 (11.0)	47 (11.0)	1 (5.0)	
Olive View‐UCLA Medical Center—no. (%)	44 (10.0)	42 (10.0)	2 (10.0)	
Truman Medical Center—no. (%)	39 (9.0)	35 (8.0)	4 (19.0)	
**Demographics**				
Age (years)—mean (SD)	43.2 (16.0)	43.4 (16.1)	39.6 (14.2)	0.25
Female sex—no. (%)	257 (57.6)	245 (57.7)	12 (57.1)	0.57
**Race**				0.31
White race—no. (%)	120 (27.0)	116 (27.0)	4 (19.0)	
Black race—no. (%)	223 (50.0)	214 (50.0)	9 (43.0)	
Asian race—no. (%)	82 (18.0)	75 (18.0)	7 (33.0)	
Other race—no. (%)	21 (5.0)	20 (5.0)	1 (5.0)	
Hispanic ethnicity—no. (%)	92 (20.8)	89 (21.1)	3 (14.3)	0.59
**Underlying health conditions**				
Health conditions—median [IQR]	0 [0, 1]	0 [0, 1]	0 [0, 1]	0.49
Chronic lung diseases—no. (%)	124 (28.0)	118 (28.0)	6 (28.6)	0.95
Cardiovascular diseases—no. (%)	64 (14.3)	61 (14.5)	3 (15.0)	0.95
Immunocompromised (including HIV/AIDS)—no. (%)	60 (13.5)	58 (13.7)	2 (9.5)	0.59
**Disease severity indicators**				
NEWS—median [IQR]	2 [1,3]	2 [1,3]	2 [1,3]	0.77
Pneumonia—no. (%)	41 (9.2)	38 (8.9)	3 (14.3)	0.43
Hospital admission—no. (%)	93 (20.9)	88 (20.7)	5 (23.8)	0.73
Hospital length of stay—median [IQR]	3 [1, 6]	3 [1, 6]	2 [1, 3]	0.37
Intensive care unit admission—no. (%)	9 (2.0)	9 (2.1)	0 (0.0)	0.65
Oxygenation—no. (%)	46 (10.3)	44 (10.4)	2 (9.5)	0.90
Deaths—no. (%)	2 (0.5)	2 (0.5)	0 (0)	0.91

Abbreviations: AIDS, acquired immunodeficiency syndrome; HIV, human immunodeficiency virus; NEWS, National Early Warning Score for acutely ill patients; IQR, interquartile range; SD, standard deviation of the mean.

^*^ *P* values for comparisons by coinfection status based on Fisher exact test and Pearson's Chi‐square for binary variables (2 × 2); Wilcoxon rank‐sum test for ordinal scores; *t* test adjusted for unequal variance for age differences.

The mean age of influenza B cases was 43.2 (SD = 16.0) years and females represented 57.6% (257/446) of the patient population. The gender distribution was similar among mono‐ and coinfections. Half of the study population (50.0% [223/446]) were Black, followed by White (26.9% [120/446]) and Asian (18.4% [82/446]). Most Asian participants were recruited at CGMH (95.1% [78/82]). Hispanic or Latin ethnicities constituted one fifth of participants (20.6% [92/446]). Patients had a median of 0 (interquartile range [IQR]: 0–1) underlying health conditions. The most prevalent comorbidities were chronic lung diseases (CLD), present in 27.8% (124/446) of cases, followed by cardiovascular diseases, which were present in 14.3% (64/446) of cases.

Table 1 also summarizes disease severity characteristics. The median NEWS was 2 (IQR: 1–3), indicating low clinical risk upon triage. Of those enrolled, 20.9% (93/446) were admitted to a hospital. The relative frequency of pneumonia was 92 per 1000 (41/446) patients, and 10.3% (46/446) patients required oxygen supplementation. Two of the admitted patients died (2.2% [2/93]), demonstrating an overall mortality rate of 4.5 per 1000 (2/446) patients.

None of the participants' demographic and health characteristics indicated statistically significant differences when comparing those individuals with coinfections to those with influenza B infections alone (Table 1). The NEWS and underlying health conditions showed the strongest unadjusted associations with the two severity outcomes: hospitalization and pneumonia.

### 3.2 Models for hospitalization

Adjusted models for estimating the odds of clinical outcomes by severity predictors are shown in Table 2. Presence of coinfections demonstrated the strongest effect size for hospitalization (OR = 2.99 [1.14–7.85], *p* = 0.026) independent of other predictors in the fitted multivariable model. The odds of hospitalization increased by 49% (OR = 1.49 [1.37–1.62], *p* < 0.001) per one‐unit increase in the NEWS in the main adjusted model. Heterogeneity across race was high with Asian participants showing an 85% decrease in odds of hospitalization compared to White participants (OR = 0.15 [0.07–0.32]; *p* < 0.001).

**TABLE 2:** Main adjusted models^a^ for estimating the odds of hospitalization and pneumonia by severity predictors

Model parameter	Hospitalization			Pneumonia		
	Odds ratio^b^	95% CI	*P* value	Odds ratio^b^	95% CI	*P* value
**Demographics**						
Age (per year)	1.04	1.03, 1.05	**<0.001**	1.02	1.01, 1.03	**<0.001**
Female vs. male	0.62	0.47, 0.80	**<0.001**	1.03	0.65, 1.64	0.907
Race/ethnicity						
Black vs. White	0.51	0.22, 1.22	0.131	0.73	0.57, 0.93	**0.011**
Asian vs. White	0.15	0.07, 0.32	**<0.001**	3.22	2.59, 3.99	**<0.001**
Other vs. White	0.86	0.42, 1.73	0.666	2.44	1.53, 3.90	**<0.001**
Hispanic/Latino vs. non‐Hispanic/Latino	0.49	0.31, 0.78	**0.003**	0.24	0.03, 1.76	0.159
**Coinfection**						
Influenza vs. influenza + coinfection	2.99	1.14, 7.85	**0.026**	2.27	1.25, 4.09	**0.007**
**Disease severity indicators**						
Underlying health conditions	2.18	1.65, 2.89	**<0.001**	3.21	1.76, 5.83	**<0.001**
NEWS	1.49	1.37, 1.62	**<0.001**	6.10	3.60, 10.32	**<0.001**

Abbreviations: 95% CI, 95% confidence interval; NEWS, National Early Warning Score for acutely ill patients.

^a^ Main models were adjusted for all variables in table.

^**b**^ Model estimates for odds ratios and 95 CI were based on multivariable logistic regressions using robust variance estimates^27^ adjusted for clustering by study site.

**FIGURE 2:**
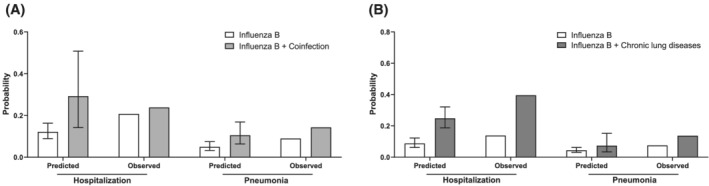
Observed and predicted adjusted probabilities (including 95% confidence intervals) for hospitalization and pneumonia diagnosis comparing patients with influenza B (white bars) and patients with influenza B coinfections with other respiratory pathogens (light gray bars; panel (A)) and comparing patients with influenza B and chronic lung diseases (dark gray bars; panel (B)). Adjusted probabilities by coinfection and chronic lung disease status were predicted from the fitted logistic regression models for the two severity outcomes, holding other predictors constant at their mean. Thin bars represent 95% confidence intervals

The adjusted probability for hospitalization among influenza B patients with coinfections (*P* = 0.29 [95% CI: 0.14–0.51]) was more than twice as high as those with influenza B monoinfections (*P* = 0.12 [0.09–0.16], Figure 2A).

### 3.3 Models for pneumonia diagnosis

Having a coinfection was statistically significantly associated with pneumonia (*p* = 0.007), increasing the odds of being diagnosed with pneumonia by more than two‐fold (OR = 2.27 [1.25–4.09]), adjusted for other predictors. An increased NEWS score (NEWS ≥ 5) resulted in a more than six‐fold increased odds (OR = 6.10 [3.60–10.32], *p* < 0.001), adjusted for other severity predictors. In contrast to the trends observed for hospitalizations, Asian race was strongly associated with increased odds of being diagnosed with pneumonia (OR = 3.22 [2.59–3.99], *p* < 0.001), while the odds of pneumonia were decreased among Black participants (OR = 0.73 [0.57–0.93], *p* = 0.011).

The predicted probability of being diagnosed with pneumonia was twice as high amongst influenza B patients with coinfections (*P* = 0.10 [0.06–0.17]) compared to influenza B patients with monoinfection (*P* = 0.05 [0.03–0.07], Figure 2A).

### 3.4 Interactions with coinfection status

All assessed interactions between coinfection status and other predictors yielded statistically nonsignificant results across the two severity outcome models.

### 3.5 Secondary analysis for chronic lung diseases

**FIGURE 3:**
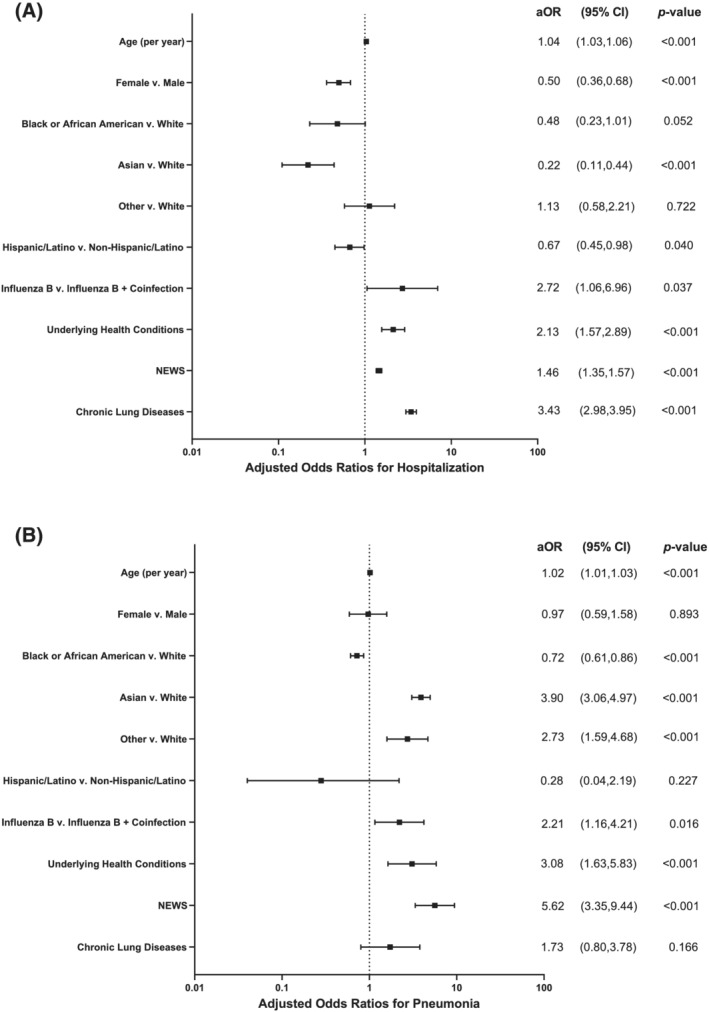
Forest plots for adjusted odds ratios (including 95% confidence intervals and *p* values) by severity predictor for (A) hospitalization and (B) pneumonia diagnosis. Adjusted odds ratios were estimated using the multivariable logistic regression models for the two severity outcomes. OR, odds ratio; 95% CI, 95% confidence interval; NEWS, National Early Warning Score for acutely ill patients

In‐depth analyses assessing the effect of specific comorbidities found that CLD was the strongest individual predictor variable for hospitalization. Having CLD increased the adjusted odds of hospitalization by more than three‐fold (OR = 3.43 [2.98–3.95], *p* < 0.001) but CLD did not independently affect the adjusted odds of being diagnosed with pneumonia (OR = 1.73 [0.80–3.78], *p* = 0.166; Figure 3). Including CLD as an additional covariate in the main models for disease severity attenuated the effect of coinfections on both hospitalization and pneumonia diagnosis. However, having a coinfection still independently increased the odds of hospitalization or pneumonia diagnosis by 172% (OR = 2.72 [1.06–6.96], *p* = 0.037) and 121% (OR = 2.21 [1.16–4.21], *p* = 0.016), respectively.

Patients with CLD were predicted to be more than twice as likely to be admitted to a hospital (*P* = 0.25 [0.19–0.32] vs. no CLD: *P* = 0.09 [0.06–0.12]), whereas CLD minimally increased the probability of a pneumonia diagnosis (*P* = 0.07 [0.03–0.15] vs. no CLD: *P* = 0.04 [0.03–0.06], Figure 2B).

## 4 DISCUSSION

In this large sample of individuals infected with influenza B (across 5 large hospitals and multiple influenza seasons), we found that having an upper viral and/or bacterial coinfection was strongly associated with more severe disease (as measured by either hospitalization or pneumonia). Having chronic lung diseases (CLD) was independently associated with hospitalization but did not increase the likelihood of being diagnosed with pneumonia.

Global influenza surveillance^1^, ^2^, ^31^ and multiple scientific reviews^5^, ^7^, ^17^ have found that seasonal influenza B has a substantial public health impact, accounting for up to 25–50% of the annual influenza burden.^1^, ^2^, ^32^, ^33^ The majority of studies describing the frequency and severity of influenza B, however, have been conducted in pediatric and/or hospitalized populations.^5^, ^34^, ^35^, ^36^ The impact of influenza B coinfections is largely demonstrated through case series and other reports,^37^, ^38^, ^39^ while systematically collected data are mostly absent. Our study reduces this evidence gap by showing an increased risk of severe disease amongst those with coinfections in an undifferentiated ambulatory population. While low clinical severity was observed, something common among patients reporting to EDs,^40^ this study underscores the potential disease severity of influenza B infections.

Whereas comorbidities represent established risk factors for severe complications associated with influenza A,^2^ investigations of the effects of specific comorbidities on disease severity are lacking for influenza B. In our study, CLD were the most common comorbidities and demonstrated the strongest effect for hospitalization, independent of other comorbidities. Given this high prevalence and strong effect of CLD on disease severity, it is important to identify CLD among influenza B patients as our study demonstrates its importance as a risk factor in understanding the clinical burden and complications of influenza B.

Additionally, we found unexpected heterogeneity in the risk of severe disease by race/ethnicity as Asians exhibited a strongly reduced susceptibility for hospitalization while being Asian was associated with an increased risk for developing pneumonia. As these effects were mostly driven by the Asian subpopulation in Taiwan, this finding may be influenced by detection bias due to differences in practices of admitting patients and diagnosing pneumonia between US and Taiwanese hospitals. However, previous studies have documented geographic variation in the burden of influenza B,^33^, ^41^, ^42^, ^43^ especially across Asia with Taiwan frequently reporting influenza B circulation.^33^, ^42^, ^44^ While these effects should be interpreted with caution, this finding underscores the importance of recruiting diverse populations in future clinical research on influenza B to elucidate the role of race/ethnicity and geographic origin in disease severity.

There are several limitations associated with this study. First, this was a secondary analysis of a biorepository and clinical dataset, which relied on either availability of residual specimens and/or prospective enrollments (Taiwan). Accordingly, potential biases may exist. However, given that the methods and protocols for sampling were well‐established at all sites and numbers of samples were robust across seasons, we do not believe that this was a major problem. A second major limitation is the relatively small number of coinfections (*n* = 21), which likely reduced the statistical power of our models. This scarcity of coinfection data is common in influenza B research^33^, ^39^ and represents a hindrance to assessing the impact of coinfections on disease severity as well as distinguishing the impact of individual pathogens as coinfections. This underscores the need to expand data collection on influenza B and coinfections to allow studies on larger patient cohorts. Third, we faced challenges in analyzing the protective effect of influenza vaccination. Varying definitions of patients' vaccination status over time and across study sites permitted a reliable assessment of vaccine coverage and estimating its effect on disease severity. While this study was not designed to estimate vaccine effectiveness, it remains crucial to examine the impact of vaccination on disease severity associated with influenza B infections in future research.

Our study highlights the importance and need for ongoing and more advanced research on influenza B. Future studies should include next‐generation sequencing to evaluate epidemiologic and clinical interactions between influenza A and B subtypes. We did not subtype influenza B samples and could accordingly not distinguish differences in disease severity between lineages. Previous research has shown age differences between patients infected with Yamagata and Victoria viruses^41^, ^45^, ^46^ and indicated influenza B Victoria to be associated with severe disease more frequently.^46^ Future influenza research should also consider emerging infectious diseases as coinfections. The COVID‐19 pandemic^47^ represents a striking example of a rapidly spreading respiratory pathogen, which should be tested for as a coinfection,^48^, ^49^ especially due to its co‐occurrence during the influenza season.^50^ Our understanding of the impact of coinfections among patients is not limited by our research capacities, but the absence of viral sequence data to conduct such research. This could be changed by using new multiplex testing technologies for respiratory pathogens and expanding genomic surveillance nationally and globally.

In conclusion, biomedical research has been focused on influenza A, resulting in substantial knowledge gaps concerning the epidemiology and pathogenesis associated with influenza B, especially regarding coinfections.^5^, ^7^, ^41^ Our study helps to improve understanding of these gaps. We identified coinfections and chronic lung diseases as the most important risk factors for severe disease complications associated with influenza B. Despite representing one of the largest epidemiologic analyses on disease severity of influenza B in association with coinfections and comorbidities to date, our results require further investigation and confirmation.

## ETHICS STATEMENT

The parent studies underlying this analysis were reviewed and approved by all participating institutions' Institutional Review Boards (IRB). Johns Hopkins University School of Medicine IRB approved protocols: IRB00135664, IRB00041233, IRB00141101, IRB00052743, and IRB00091667.

## PATIENT CONSENT STATEMENT

All participants in the parent studies consented to have their samples made available and used for future research.

## PERMISSION TO REPRODUCE MATERIALS FROM OTHER SOURCES

Does not apply to this study. The authors declare that this manuscript does not contain any previously published material (including figures/diagrams, or short extracts, or content taken from websites), and all figures and tables are original.

## AUTHOR CONTRIBUTIONS

**Alexander Zapf:** Conceptualization; formal analysis. **Justin Hardick:** Data curation; formal analysis; methodology. **Breana McBryde:** Data curation; investigation; project administration. **Lauren Sauer:** Investigation; supervision. **Katherine Fenstermacher:** Data curation; project administration. **Erin P. Ricketts:** Data curation; investigation; project administration. **Yi‐Chin Lin:** Data curation; investigation; project administration. **Kuan‐Fu Chen:** Investigation; supervision. **Yu‐Hsiang Hsieh:** Formal analysis; investigation; supervision. **Andrea Dugas:** Funding acquisition; investigation; project administration. **Kathryn Shaw‐Saliba:** Conceptualization; formal analysis; investigation; supervision; visualization. **Andrew Pekosz:** Investigation; supervision. **charlotte gaydos:** Investigation; supervision. **Richard Rothman:** Investigation; supervision.

## CONFLICT OF INTEREST

All authors declare that they have no conflict of interest.

### PEER REVIEW

The peer review history for this article is available at https://publons.com/publon/10.1111/irv.12907.

## Data Availability

Data are available on request due to privacy/ethical restrictions: The data that support the findings of this study are available on request from the corresponding author. The data are not publicly available due to privacy or ethical restrictions. Some of the supporting data represent protected health information (PHI) under the HIPAA Privacy Rule and therefore cannot be shared but requestors could have access to the diagnostic test results.

## References

[1] World Health Organization . Global influenza programme. Estimate of influenza deaths due to respiratory disease. Accessed August 20, 2021. https://www.who.int/teams/global-influenza-programme/surveillance-and-monitoring/burden-of-disease

[2] Centers for Disease Control and Prevention . Disease burden of influenza. Accessed August 20, 2021. https://www.cdc.gov/flu/about/burden/index.html

[3] Centers for Disease Prevention and Control . About flu. Accessed August 20, 2021. https://www.cdc.gov/flu/about/index.html

[4] Alexopulos N. Characteristics of pandemic pathogens. Johns Hopkins Center for Health Security August 20, 2021. http://www.centerforhealthsecurity.org/newsroom/news_releases/2018-05-10_characteristics-of-pandemic-pathogens.html

[5] Glezen PW , Schmier JK , Kuehn CM , Ryan KJ , Oxford J . The burden of influenza B: a structured literature review. Am J Public Health. 2013;103(3):e43‐e51. 10.2105/AJPH.2012.301137 PMC3673513 23327249

[6] Neumann G , Noda T , Kawaoka Y . Emergence and pandemic potential of swine‐origin H1N1 influenza virus. Nature. 2009;459(7249):931‐939. 10.1038/nature08157 19525932 PMC2873852

[7] van de Sandt CE , Bodewes R , Rimmelzwaan GF , de Vries RD . Influenza B viruses: not to be discounted. Future Microbiol. 2015;10(9):1447‐1465. 10.2217/fmb.15.65 26357957

[8] Valesano AL , Fitzsimmons WJ , McCrone JT , et al. Influenza b viruses exhibit lower within‐host diversity than influenza a viruses in human hosts. J Virol. 94(5):e01710‐e01719. 10.1128/JVI.01710-19 PMC7022338 31801858

[9] Baselga‐Moreno V , Trushakova S , McNeil S , et al. Influenza epidemiology and influenza vaccine effectiveness during the 2016–2017 season in the Global Influenza Hospital Surveillance Network (GIHSN). BMC Public Health. 2019;19(1):487. 10.1186/s12889-019-6713-5 31046725 PMC6498567

[10] Caini S , Spreeuwenberg P , Kusznierz GF , et al. Distribution of influenza virus types by age using case‐based global surveillance data from twenty‐nine countries, 1999‐2014. BMC Infect Dis. 2018;18(1):269. 10.1186/s12879-018-3181-y 29884140 PMC5994061

[11] Coleman BL , Fadel SA , Fitzpatrick T , Thomas S‐M . Risk factors for serious outcomes associated with influenza illness in high‐ versus low‐ and middle‐income countries: systematic literature review and meta‐analysis. Influenza Other Respi Viruses. 2018;12(1):22‐29. 10.1111/irv.12504 PMC5818335 29197154

[12] Mertz D , Kim TH , Johnstone J , et al. Populations at risk for severe or complicated influenza illness: systematic review and meta‐analysis. BMJ. 2013;347. 10.1136/bmj.f5061 PMC3805492 23974637

[13] Mertz D , Lo CK‐F , Lytvyn L , et al. Pregnancy as a risk factor for severe influenza infection: an individual participant data meta‐analysis. BMC Infect Dis. 2019;19(1):683. 10.1186/s12879-019-4318-3 31375073 PMC6679491

[14] Brundage JF . Interactions between influenza and bacterial respiratory pathogens: implications for pandemic preparedness. Lancet Infect Dis. 2006;6(5):303‐312. 10.1016/S1473-3099(06)70466-2 16631551 PMC7106411

[15] Brundage JF , Shanks GD . Deaths from bacterial pneumonia during 1918–19 influenza pandemic. Emerg Infect Dis J. 2008;14(8):1193‐1199. 10.3201/eid1408.071313 PMC2600384 18680641

[16] Morris DE , Cleary DW , Clarke SC . Secondary bacterial infections associated with influenza pandemics. Front Microbiol. 2017;8:1041. 10.3389/fmicb.2017.01041 28690590 PMC5481322

[17] Glezen PW . Editorial commentary: changing epidemiology of influenza b virus. Clin Infect Dis. 2014;59(11):1525‐1526. 10.1093/cid/ciu668 25139967

[18] Dugas AF , Hsieh Y‐H , Lovecchio F , et al. Derivation and validation of a clinical decision guideline for influenza testing in four U.S emergency departments. Clin Infect Dis. 70(1):49‐58. 10.1093/cid/ciz171 30843056

[19] Centers for Disease Control and Prevention . People at High Risk of Flu Complications. Accessed August 20, 2021. https://www.cdc.gov/flu/highrisk/index.htm

[20] McGinley A , Pearse RM . A national early warning score for acutely ill patients. BMJ. 2012;345(aug08 1):e5310. 10.1136/bmj.e5310 22875955

[21] Bilben B , Grandal L , Sovik S . National Early Warning Score (NEWS) as an emergency department predictor of disease severity and 90‐day survival in the acutely dyspneic patient—a prospective observational study. Scand J Trauma Resusc Emerg Med. 2016;24(1):80. 10.1186/s13049-016-0273-9 27250249 PMC4890514

[22] Caini S , Kroneman M , Wiegers T , El Guerche‐Séblain C , Paget J . Clinical characteristics and severity of influenza infections by virus type, subtype, and lineage: a systematic literature review. Influenza Other Respi Viruses. 2018;12(6):780‐792. 10.1111/irv.12575 PMC6185883 29858537

[23] Thompson WW , Shay DK , Weintraub E , et al. Influenza‐associated hospitalizations in the United States. JAMA. 2004;292(11):1333‐1340. 10.1001/jama.292.11.1333 15367555

[24] Shapiro SS , Wilk MB . An analysis of variance test for normality (complete samples)†. Biometrika. 1965;52(3–4):591‐611. 10.1093/biomet/52.3-4.591

[25] Lindsey C , Sheather S . GVSELECT: Stata module to perform best subsets variable selection. Stat Softw Compon August 20, 2021. https://ideas.repec.org/c/boc/bocode/s457816.html

[26] Smith GB , Prytherch DR , Meredith P , Schmidt PE , Featherstone PI . The ability of the National Early Warning Score (NEWS) to discriminate patients at risk of early cardiac arrest, unanticipated intensive care unit admission, and death. Resuscitation. 2013;84(4):465‐470. 10.1016/j.resuscitation.2012.12.016 23295778

[27] Rogers W . Regression standard errors in clustered samples. Stata Tech Bull. 1994;3(13). https://EconPapers.repec.org/RePEc:tsj:stbull:y:1994:v:3:i:13:sg17

[28] Huber PJ . The behavior of maximum likelihood estimates under nonstandard conditions. 13. Berkeley Symposium on Mathematical Statistics and Probability, 1967:221‐233. Available online: http://www.personal.psu.edu/~aza12/huber_s08.pd

[29] White H . A heteroskedasticity‐consistent covariance matrix estimator and a direct test for heteroskedasticity. Econometrica. 1980;48(4):817‐838. 10.2307/1912934

[30] Hosmer DW , Lemeshow S . Goodness of fit tests for the multiple logistic regression model. Commun Stat—Theory Methods. 1980;9(10):1043‐1069. 10.1080/03610928008827941

[31] de Barros ENC , Cintra O , Rossetto E , Freitas L , Colindres R . Patterns of influenza B circulation in Brazil and its relevance to seasonal vaccine composition. Braz J Infect Dis Off Publ Braz Soc Infect Dis. 2016;20(1):81‐90. 10.1016/j.bjid.2015.09.009 PMC7110561 26626166

[32] Caini S , El‐Guerche Séblain C , Ciblak MA , Paget J . Epidemiology of seasonal influenza in the Middle East and North Africa regions, 2010‐2016: circulating influenza A and B viruses and spatial timing of epidemics. Influenza Other Respi Viruses. 2018;12(3):344‐352. 10.1111/irv.12544 PMC5907816 29405575

[33] Caini S , Huang QS , Ciblak MA , et al. Epidemiological and virological characteristics of influenza B: results of the global influenza B study. Influenza Other Respi Viruses. 2015;9(Suppl 1):3‐12. 10.1111/irv.12319 PMC4549097 26256290

[34] Bender JM , Ampofo K , Gesteland P , et al. Influenza virus infection in infants less than three months of age. Pediatr Infect Dis J. 2010;29(1). https://journals.lww.com/pidj/Fulltext/2010/01000/Influenza_Virus_Infection_in_Infants_Less_Than.5.aspx 10.1097/INF.0b013e3181b4b950 19915513

[35] Iskander M , Kesson A , Dwyer D , et al. The burden of influenza in children under 5 years admitted to the Children's Hospital at Westmead in the winter of 2006. J Paediatr Child Health. 2009;45(12):698‐703. 10.1111/j.1440-1754.2009.01597.x 19895431

[36] Wong KK , Jain S , Blanton L , et al. Influenza‐associated pediatric deaths in the United States, 2004‐2012. Pediatrics. 2013;132(5):796‐804. 10.1542/peds.2013-1493 24167165 PMC6027595

[37] Shah MM , Hsiao EI , Kirsch CM , Gohil A , Narasimhan S , Stevens DA . Invasive pulmonary aspergillosis and influenza co‐infection in immunocompetent hosts: case reports and review of the literature. Diagn Microbiol Infect Dis. 2018;91(2):147‐152. 10.1016/j.diagmicrobio.2018.01.014 29454654 PMC5970059

[38] Spoto S , Valeriani E , Locorriere L , et al. Influenza B virus infection complicated by life‐threatening pericarditis: a unique case‐report and literature review. BMC Infect Dis. 2019;19(1):40. 10.1186/s12879-018-3606-7 30630424 PMC6327550

[39] Stefanska I , Romanowska M , Donevski S , Gawryluk D , Brydak LB . Co‐infections with influenza and other respiratory viruses. In: Pokorski M , ed. Respiratory Regulation—The Molecular Approach. Springer Netherlands; 2013:291‐301. 10.1007/978-94-007-4549-0_36 PMC7120114 22836647

[40] Walsh EE , McConnochie KM , Long CE , Hall CB . Severity of respiratory syncytial virus infection is related to virus strain. J Infect Dis. 1997;175(4):814‐820. 10.1086/513976 9086135

[41] Caini S , Kusznierz G , Garate VV , et al. The epidemiological signature of influenza B virus and its B/Victoria and B/Yamagata lineages in the 21st century. PLoS ONE. 2019;14(9):e0222381. 10.1371/journal.pone.0222381 31513690 PMC6742362

[42] Cowling BJ , Caini S , Chotpitayasunondh T , et al. Influenza in the Asia‐Pacific region: findings and recommendations from the global influenza initiative. Vaccine. 2017;35(6):856‐864. 10.1016/j.vaccine.2016.12.064 28081970

[43] Tafalla M , Buijssen M , Geets R , Vonk N‐SM . A comprehensive review of the epidemiology and disease burden of Influenza B in 9 European countries. Hum Vaccin Immunother. 2016;12(4):993‐1002. 10.1080/21645515.2015.1111494 26890005 PMC4962970

[44] Jennings L , Huang QS , Barr I , et al. Literature review of the epidemiology of influenza B disease in 15 countries in the Asia‐Pacific region. Influenza Other Respi Viruses. 2018;12(3):383‐411. 10.1111/irv.12522 PMC5907823 29127742

[45] Barr IG , Vijaykrishna D , Sullivan SG . Differential age susceptibility to influenza B/Victoria lineage viruses in the 2015 Australian influenza season. Eurosurveillance. 2016;21(4):30118. 10.2807/1560-7917.ES.2016.21.4.30118 26848118

[46] Horthongkham N , Athipanyasilp N , Pattama A , et al. Epidemiological, clinical and virological characteristics of influenza B virus from patients at the hospital tertiary care units in Bangkok during 2011‐2014. PLoS ONE. 2016;11(7):e0158244. 10.1371/journal.pone.0158244 27387488 PMC4936745

[47] Centers for Disease Control and Prevention Coronavirus Disease 2019 (COVID‐19). Accessed August 20, 2021. https://www.cdc.gov/coronavirus/2019-ncov/index.html

[48] Kim D , Quinn J , Pinsky B , Shah NH , Brown I . Rates of co‐infection between SARS‐CoV‐2 and other respiratory pathogens. JAMA. 2020;323(20):2085‐2086. 10.1001/jama.2020.6266 32293646 PMC7160748

[49] Lansbury L , Lim B , Baskaran V , Lim WS . Co‐infections in people with COVID‐19: a systematic review and meta‐analysis. J Infect. 2020;2:266‐275. 10.1016/j.jinf.2020.05.046 PMC7255350 32473235

[50] Wu D , Lu J , Ma X , et al. Coinfection of influenza virus and severe acute respiratory syndrome coronavirus 2 (SARS‐COV‐2). Pediatr Infect Dis J. 2020;39(6):e79. 10.1097/INF.0000000000002688 PMC7258765 32287051

